# Focal epilepsy with ictal abdominal pain: a case report

**DOI:** 10.1186/1824-7288-39-76

**Published:** 2013-12-09

**Authors:** Caterina Cerminara, Nadia El Malhany, Denis Roberto, Paolo Curatolo

**Affiliations:** 1 Department of Neuroscience, Paediatric Neurology Unit, “Tor Vergata” University of Rome, Viale Oxford, 81, 00133 Rome, Italy

## Abstract

Focal epilepsy with ictal abdominal pain is an unusual partial epilepsy characterized by paroxysmal episodes of abdominal or visceral pain, disturbance of awareness and electroencephalographic abnormalities. We describe a new case of ictal abdominal pain in which gastrointestinal complaints were the only manifestation of seizures and review the previously described pediatric patients. In our patient clinical findings, ictal EEG abnormalities, and a good response to antiepileptic drugs allowed us to make a diagnosis of focal epilepsy with ictal abdominal pain. This is a rare epileptic phenomenon that should be suspected in patients with unexplained paroxysmal abdominal pain and migraine-like symptoms. We suggest that, after the exclusion of more common etiologies, focal epilepsy with ictal abdominal pain should be considered in patients with paroxysmal abdominal pain and ictal EEG abnormalities.

## Introduction

Recurrent episodes of abdominal pain are common in children and adults. Several pathological conditions can lead to paroxysmal gastrointestinal symptoms, such as porphiria, cyclical vomiting, intestinal malrotation, peritoneal bands, and abdominal migraine [1]. Psychological and emotional factors may also play an important role in some patients with gastrointestinal disorders. However, in a number of patients the episodic nature of abdominal pain can be suggestive for a diagnosis of epilepsy [1]. Epileptiform EEG abnormalities, loss or alteration of consciousness, and a good response to antiepileptic drugs are other features that can lead to a diagnosis of focal epilepsy with ictal abdominal pain [2,3]. We describe one child affected by epilepsy which had recurrent and severe abdominal pain as the only manifestation of epileptic seizures.

## Case report

An 8-year-old boy was born at 39 weeks of gestation by selective cesarean section. The pregnancy was complicated by a sudden reduction in fetal heart rate. All developmental milestones were regularly achieved. There was no family history of epilepsy. The boy experienced recurrent episodes of abdominal pain since about 6 months of age. He described the pain as “a sword that pierces my belly”, localized mainly in the epigastric region and its duration varied from a few minutes (more frequently) to 1 hour, with a frequency of 5–8 episodes per day. The intensive abdominal pain was almost always associated with pallor and nausea, but not accompanied by scream or cry. The attacks were sudden in onset and had spontaneous resolution. There was no impairment of consciousness, also in longer episodes, and he never had convulsions; the paroxysms were followed by increased sleep. He underwent a negative abdominal investigation including complete blood count, stool examinations for ova and parasites, abdominal ultrasound and upper gastrointestinal endoscopy. Physical and neurological examinations were normal.

**Figure 1:**
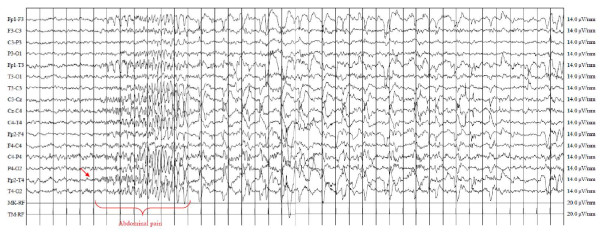
**Ictal awake EEG showing runs of rhythmic spikes and sharp waves, over the right fronto-temporal electrodes.** The onset and offset of abdominal pain are closely related to the beginning and end of the discharge.

Interictal EEG during wakefulness and sleep displayed bilateral spikes and diphasic sharp-waves localized over the temporal leads with a marked increase in frequency during drowsiness. A 24-hours EEG recording showed several bilateral synchronous and asynchronous temporal spikes during wakefulness and nocturnal sleep. At 9:30 in the morning a seizure characterized by severe abdominal pain in the epigastric region with nausea and pallor was recorded. The ictal EEG showed rhythmic spikes on the centro-temporal regions (Figure 1). Magnetic resonance imaging of the brain was normal. The patient started treatment with Carbamazepine (CBZ) (20 mg/kg/day) with a progressive decrease in seizure frequency. At the last follow-up, when he was 9-years old, he was seizure free.

## Discussion

Epigastric sensations are frequent symptoms in patients with partial epilepsy and may include abdominal pain, nausea, vomiting and hunger, and have been reported to be the most common aura in temporal lobe epilepsy [3-5]. Painful epileptic auras were reported in 4.1% of 25 patients with focal epilepsy by Nair et al. [6]. Abdominal pain was present in 5% of all abdominal auras in temporal lobe epilepsy and 50% in frontal lobe epilepsy [6]. However, gastrointestinal complaints, in particular abdominal pain, may be the only manifestation of epileptic activity [1,3,4,7]. Unexplained paroxysmal gastrointestinal complaints, impairment of consciousness, and focal abnormal EEG are the main criteria to establish a diagnosis of focal epilepsy with ictal abdominal pain, but not all the criteria need to be present in each case [2,3,6]. In addition, a variety of migraine-like disturbances such as nausea, headache, dizziness, and visual hallucinations may be associated with pain during the attacks [4]*.* When the migraine-like symptoms are present it is often difficult to differentiate focal epilepsy with ictal abdominal pain from migraine or other neurological disorders, such as Panayiotopoulos syndrome. The abrupt onset, the spontaneous resolution, and the relatively short duration of episodes may be helpful for a correct and early diagnosis of focal epilepsy with ictal abdominal pain. Another helpful distinguishing feature of epilepsy with severe abdominal pain could be the localization of ictal pain, that is most commonly periumbilical or upper abdominal and rarely spreads to involve other body parts, such as in our patient [1-4].

EEG abnormalities have been reported in most patients with focal epilepsy and ictal abdominal pain [1,4]. Few reports described ictal EEGs: during the seizure the EEG often shows a runs of high voltage slow waves and generalized spike and wave discharges [2-4,8]. In our patient, 24-hours EEG was suggestive of a focal onset, as in two reports that showed clear focal EEG changes over the left hemisphere during an episode of abdominal pain [9,10]. Table 1 shows the clinical characteristics of our patient and the previous pediatric cases described in literature (Table 1) [1-5,7-13].

**Table 1:** Clinical characteristics of reported cases of abdominal epilepsy in pediatric population and in our patient

**Patient number**	**Age**	**Sex**	**Gastrointestinal symptoms**	**Other non-gastrointestinal symptoms**	**Episode duration**	**EEG**	**Treatment**	**Outcome**
**Zdravescka N. et al.**^**1**^	14	F	Colicky epigastric pain, nausea, vomiting and diarrhea	Pallor, dizziness	10-30 minutes	Spikes, sharp waves over the right central and temporal regions with secondary generalization	Carbamazepine	Seizure free
**Franzon RC et al.**^**2**^	6	F	Abdominal pain	Disturbed awareness, occasional generalized tonic-clonic seizures	Seconds to minutes	Spikes and slow waves over left temporal area	Anticonvulsants, surgical resection of oligoastrocytoma	Seizure free
**Garcia-Herrero D. et al.**^**3**^	14	F	Colicky periumbilical pain	Headache, pallor, dizziness, multicolored photopsia	Second to minutes	Interictal-bursts of sharp and slow waves	Valproic acid	Near complete resolution
**Dutta SR et al.**^**4**^	15	M	Epigastric abdominal pain and vomiting	Lethargy	30 minute to hours	Right temporal focal seizure discharge with generalization	Oxcarbazepine	Seizure free
Case 1								
**Dutta SR et al.**^**4**^	13	F	Colicky periumbilical pain	NR	10-30 minutes	Generalized spikes and wave discharges	Oxcarbazepine	Seizure free
Case 2								
**Young GB et al.**^**5**^	15	F	Abdominal pain	Generalized tonic seizures	NR	Multiple independent spikes	NR	NR
**Hasan N. et al.**^**7**^	8	M	Colicky periumbilical pain, vomiting	Pallor, an episode with jerky movements in the lower limbs	10-30 minutes	Generalized paroxysmal epileptiform activity, maximum on photic stimulation	Valproic acid	Seizure free
**Siegel AM et al.**^**8**^	1	F	Crampy periumbilical pain	Occasional generalized seizures	Few seconds	Right parietal focus	NR	NR
**Mitchell WG et al.**^**9**^	6	M	Vomiting	Bad smell, fatigue	20-40 seconds	Ictal and intercritic high voltage arrhythmic delta waves, sometimes sharply contoured	Multiple antiseizure medication, than surgary and radiation (for astrocytoma)	Decreased frequency of episodes
**Douglas EF et al.**^**10**^	11	F	Paroxysmal, peri-umbilical abdominal pain	Lassitude, post-ictal sleep, fever, headache, confusion.	“Brief”	Irregular 3 Hz spike-waves activity	Phenobarbital	Seizure free
Case 1								
**Douglas EF et al.**^**10**^	5	F	Crampy, paroxysmal abdominal pain	Lethargy, post-ictal sleep	Few minutes	Episode 6–7 activity in L temporal area, burst of generalized irregularly intermixed spikes and slow waves	Phenobarbital	Lost to follow-up
Case 2								
**Douglas EF et al.**^**10**^	6	M	Paroxysmal pain	Lethargy, confusion, fever	Few minutes	Paroxysmal spike-wave activity, frontal or generalized	Anticonvulsivants	Seizure free
Case 3								
**Yingkun F**^**11**^	3	M	Abdominal pain, vomiting	Confusion, cyanosis, urinary incontinence, blindness	Few minutes	Scattered high voltage slow activity and high voltage sharp waves	Phenytoin, phenobarbital	Seizure free
Case 1								
**Yingkun F **^**11**^	16	M	Upper abdominal pain, nausea	Disturbance of consciousness	3-5 minutes	High voltage slow waves; high voltage sharp waves with hyperventilation	Phenobarbital	Seizure free
Case 2								
**Yingkun F et al.**^**11**^	11	F	Periumbilical abdominal pain	Disturbance/loss of consciousness	Minutes to hour	Bilateral high voltage spikes, complexed slow waves	Phenytoin, phenobarbital	Seizure free
Case 3								
**Singhi PD et al.**^**12**^	10	M	Periumbilical pain	Pallor, sweates, lethargy, post-ictal sleep	Few minutes	Sharp spikes, spikes and wave activity arising over the central region and becoming generalized	Phenytoin	Complete resolution
**Agrawal P. et al.**^**13**^	6	M	Colicky periumbilical pain	Lassitude, post-ictal sleep	Half an hour	Generalized slowing, right posterior spikes	Carbamazepine	Seizure free
**Our patient**	8	M	Colicky epigastric pain, nausea	Pallor	Few minutes to 1 hour	Bilateral synchronous and asynchronous spikes and diphasic sharp-waves in temporal and central area, increased during drowsiness and sleep	Carbamazepine	Seizure free

The pathophysiology of focal epilepsy with ictal abdominal pain remains unknown. Abdominal sensations reproduced by stimulating the insula and sylvian fissure, suggest that these areas may have an important role in explaining the origin of focal epilepsy with ictal abdominal pain [3]. Phan et al. [14], reported an unusual case of ictal abdominal pain occurring in the setting of parietal lobe haemorrhage and suggested a possible role of the somatosensory area in pain perception. Supplementary motor area was considered as another possible location for abdominal pain. Occasionally focal epilepsy with ictal abdominal pain has been related to brain tumors and brain disorders [2,8]. Previous reports on ictal abdominal pain have shown right parieto-occipital encephalomalacia, biparietal atrophy and bilateral perisylvian polymicrogyria [9].

In conclusion, our patient showed recurrent attacks of severe abdominal pain as the only manifestation of epileptic seizure. Focal epilepsy with ictal abdominal pain is a rare epileptic phenomenon that should be suspected in patients with unexplained paroxysmal abdominal pain and migraine-like symptoms. The correct diagnosis at the onset may be difficult to establish; in these cases prolonged EEG recordings with 24-hours monitoring must be considered to facilitate the clinical diagnosis.

## Consent

Written informed consent was obtained from the patient’s parents for the publication of this report.

## Competing interests

The authors declare that they have no financial and non-financial competing interests.

## Authors’ contributions

CC (Medical Doctor) drew the first draft with the assistance and contribution of NEM (Medical Doctor); DR (Medical Doctor) reviewed relevant articles on the literature under the supervision of PC (Director of the Department of Pediatric Neuroscience Unit); PC revised the final draft. All authors contributed to the intellectual contents and approved the final version.
